# Development of a business continuity plan and implementation of headquarters operation training considering a nuclear disaster

**DOI:** 10.3389/fpubh.2025.1612798

**Published:** 2025-06-20

**Authors:** Takakiyo Tsujiguchi, Masato Naraoka, Kanako Yamanouchi, Ryutaro Mori, Ryusei Maekawa, Ikuo Kashiwakura, Katsuhiro Ito

**Affiliations:** ^1^Hirosaki University Radiation Emergency Medicine and Cooperation Promotion Education Center for Disaster and Radiation Emergency Medicine, Hirosaki, Aomori, Japan; ^2^Advance Emergency and Critical Care Center, Hirosaki University Hospital, Hirosaki, Aomori, Japan; ^3^Graduate School of Health Sciences, Hirosaki University, Hirosaki, Aomori, Japan

**Keywords:** business continuity plan, disaster response training, hospital disaster management, radiation emergency medicine, nuclear disaster response

## Abstract

Hospitals play a critical role in protecting the lives and health of residents during disasters, making the development and effective implementation of business continuity plans (BCPs) essential. During nuclear disasters, it is particularly important to establish a system capable of specialized responses. This study reports on the efforts of our hospital, designated as an Advanced Radiation Emergency Medical Support Center, to develop a BCP that addresses both natural and nuclear disasters and implement operation training at the headquarters. In 2022, nuclear disaster response was incorporated into the existing BCP, and in 2023, training exercises were conducted based on compound disaster scenarios. During the training, participants practiced information sharing and decision-making through simulated communication while performing their designated roles. The post-training review identified issues such as a limited understanding of nuclear disaster-specific terminology, a lack of established procedures for dispatching radiological technologists, and an absence of media response guidelines. These findings demonstrated the practical value of BCP development and training implementation for enhancing preparedness against various types of disasters, and provide insights for the continuous improvement of hospital disaster response systems and educational frameworks.

## 1 Introduction

Hospitals are essential institutions in the society, responsible for safeguarding the lives and health of disaster victims. However, disaster preparedness among hospitals in Japan remains inadequate, and recent large-scale disasters have revealed significant vulnerabilities in hospital functions (1–3). The concept of a business continuity plan (BCP) was introduced in Japan in the 2000s, primarily in the context of information security and infectious disease control. Nonetheless, the importance of hospital-specific BCPs was not fully recognized until after the Great East Japan Earthquake (GEJET) in 2011 (1, 4, 5). Although many hospitals withstood structural collapse during the GEJET, they experienced functional failures and could not deliver sufficient medical care. This led to a substantial number of disaster-related deaths, many of which were preventable with proper medical systems in place. In response to these shortcomings, designated disaster base hospitals in Japan were required not only to ensure seismic resilience and improve facilities but also formulate BCP and conduct regular operational training (1). This highlights the growing awareness of hospitals’ critical roles in disaster response and the need for structured, scenario-based preparedness to ensure the continuity of essential medical services during crises.

Following the Fukushima Daiichi nuclear power plant accident during the GEJET, the country designated nuclear emergency core hospitals and Advanced Radiation Emergency Medical Support Centers nationwide. These institutions have been working on the development of systems for treating radiation-exposed and contaminated patients, as well as mutual support strategies (6). In response, the government issued guidelines encouraging these institutions to develop BCP and conduct related training specifically considering nuclear disasters at nearby nuclear facilities (7). However, reports on the extent to which institutions involved in nuclear disaster medicine nationwide have developed BCPs and the types of training they have implemented remain scarce.

The authors’ institution was designated by the national government as an Advanced Radiation Emergency Medical Support Center and is one of the institutions in Japan required to develop a BCP and conduct related training. In 2022, the hospital developed a BCP for a nuclear disaster scenario in the Aomori Prefecture, Japan, where the hospital is located. Since then, the hospital has conducted operational drills at the disaster response headquarters based on the assumption of a combined natural and nuclear disaster event.

The objective of this study is to provide a detailed report on the development of a hospital Business Continuity Plan (BCP) for nuclear disaster scenarios and the implementation of operational drills at the disaster response headquarters. By identifying issues and challenges revealed during these drills, the study aims to offer concrete insights to enhance hospital disaster preparedness, particularly regarding the effectiveness of BCPs in complex disaster situations involving both natural and nuclear events. Furthermore, this research is expected to contribute valuable information for planning future disaster training and revising BCPs, serving as a model for similar institutions domestically and internationally. Ultimately, these efforts seek to improve hospitals’ disaster response capabilities and ensure the continuity of medical care for affected populations.

## 2 Situation regarding the development of the hospital’s BCP

### 2.1 Situation regarding the hospital’s designation for disaster medical care and the development of a BCP for natural disasters

Our hospital is located in the Tsugaru region of the Aomori Prefecture in northern Japan and was designated as a disaster base hospital by the Aomori Prefecture in 2015. In 2018, the hospital developed a BCP outlining the system and response strategies for potential natural disasters in the Tsugaru region and internal emergencies such as fires. Since then, the hospital has conducted annual drills to receive several injured and ill patients, as well as disaster response headquarters’ operation training.

### 2.2 Background and content of the development of the BCP assuming a nuclear disaster

In 2015, our hospital was designated as an Advanced Radiation Emergency Medical Support Center by the Japan Nuclear Regulation Authority and became a key institution for radiation emergency medical care in Japan (8). The Aomori Prefecture, where the hospital is located, houses numerous nuclear facilities, including commercial nuclear power plants and nuclear fuel intermediate storage facilities. Although the hospital is located approximately 100 km from these nuclear facilities, the risk of elevated ambient radiation levels in the vicinity of the hospital in the event of a nuclear disaster involving the release of radioactive materials is low. However, hospitals are still required to provide emergency medical care for radiation-exposed and radiation-contaminated patients, offer radiation consultation services to evacuees, and dispatch radiation specialists to conduct contamination assessments. Therefore, it has become necessary to incorporate nuclear disaster response into the existing BCP, along with the notification and training of hospital staff.

Therefore, in 2022, our hospital revised the existing BCP to include the following elements related to nuclear disasters.

- Types of nuclear facilities in Aomori prefecture and an overview of potential accidents/disasters
- Prediction of resident evacuation during a nuclear disaster
- Criteria for establishing a nuclear disaster response division within disaster response headquarters and a communication network for internal stakeholders
- Contact information for relevant government agencies and associated companies during a nuclear disaster
- System for receiving radiation-exposed/contaminated patients, with separate patient pathways from those of regular disasters.

In incorporating these additions, a reference was made to the materials provided by Aomori Prefecture and relevant organizations.

## 3 Methods

### 3.1 Overview of the disaster response headquarters training assuming a nuclear disaster

Following the revision of the BCP, disaster response headquarters training simulating combined natural and nuclear disasters has been conducted since 2023. During the training, participants first reviewed their roles according to the BCP. Next, they verified whether the layout of the disaster response headquarters and necessary materials such as whiteboards for information consolidation and telephone lines were properly arranged. Subsequently, a one-hour practical training session was conducted. During the training, the controller made various calls, and participants focused on accurately receiving information, relaying it to relevant departments inside and outside the hospital, and ensuring effective communication to solve problems. The overall training scenario, participants, and communication training scenario for the controller are summarized in Table 1. The scenarios were developed by internal and external disaster medical experts involved in BCP creation, based on actual large-scale disaster cases. Practical training on treating radiation-exposed/contaminated patients is conducted separately by physicians, nurses, and radiological technologists from the emergency medical center. This training did not include practical medical procedures.

**Table 1:** Overview of the disaster response headquarters training assuming a nuclear disaster, and an example of the scenarios considered by trainees assigned as headquarters personnel.

Overall assumptions of the training		A magnitude of 8 earthquakes centered on an active fault located off the east coast of Aomori Prefecture, followed 3 days later by an environmental release of radioactive materials from the damaged nuclear power plant, resulting in a nuclear disaster.	
Assumption	Inside the hospital	Mass casualty patient visits: “Although 3 days have passed since the earthquake and the number of mass casualty patients has begun to decline, the hospital continues to maintain an emergency system, including the capability to conduct triage in front of the facility, in preparation for any unforeseen circumstances. Infrastructure: No problem Staffing levels are sufficient. Status of in-hospital PHS, internet connectivity, and satellite phone establishment: All systems have been established. Outpatient: Normal operation Medical equipment and materials: No problem	
	Outside the hospital	A system enabling telephone communication with external agencies has been established. Ambient radiation levels in the area surrounding the hospital remain within normal limits. Evacuation orders have been issued for up to 70,000 residents living near the nuclear facility, and approximately 10% (around 7,000 individuals) may evacuate to the area where the hospital is located. Infrastructure has been severely affected, with many areas still experiencing road damage, power outages, and water supply disruptions. Numerous evacuation shelters have been established by local governments, and a significant number of residents are currently taking refuge in these shelters.	
Participants		Healthcare professionals, including physicians, nurses, pharmacists, clinical laboratory technologists, and radiological technologists, as well as administrative-level personnel such as hospital directors and heads of nursing departments. Administrative staff of the hospital.	
An example of a telephone communication scenario provided by the training controller*		The role played by the controller	Content
		Nuclear operator	“We have a trauma patient accompanied by radioactive contamination. We would like to request the patient’s acceptance and medical care at your facility.”
		Aomori prefectural government official	“In preparation for conducting contamination screening of residents evacuating from the vicinity of the nuclear power plant, we would like to request the dispatch of several radiological technologists, if available.”
		Aomori prefectural government official	“Regarding essential hospital lifelines such as electricity, water, and medical gases, as well as medical supplies and equipment, do you have any requests for replenishment? Please assess the situation within your facility and inform us if there are any items that should be procured at the prefectural level.”
		Television broadcaster	“We have obtained information indicating that patients affected by a nuclear disaster are being treated. We would like to request access for media coverage, subject to your approval.”

*A total of 30 scenarios have been prepared for the trainees assigned as headquarters personnel to consider appropriate responses, and a portion of them is presented in this table.

### 3.2 Details of the training

The hospital conducts approximately three-hour training sessions twice a year. About 1 h is dedicated to introducing the BCP and lectures on establishing the disaster response headquarters, followed by 90 min of operational drills. The final 30 min are reserved for a debriefing session where all participants discuss issues and challenges in the BCP. Participants act as members of the disaster response headquarters, assuming roles such as information collection, liaison, and recording staff, as well as medical personnel responsible for patient information management and clinical coordination according to their professional background. Each session involves 15 participants: 3 physicians, 3 nurses, 3 medical technologists (pharmacists, radiological technologists, etc.), and 6 administrative staff. All participants have over 10 years of experience and serve as managers in their respective departments. The training is led by an in-house disaster medical expert who holds a Disaster Medical Assistance Team instructor certification.

### 3.3 Issue extraction

Post-training debriefings were conducted as free discussions to collect participants’ opinions on issues needing improvement in the BCP. Although no quantitative surveys were conducted, commonly raised issues by many participants are summarized in the Results section.

## 4 Results

**Figure 1:**
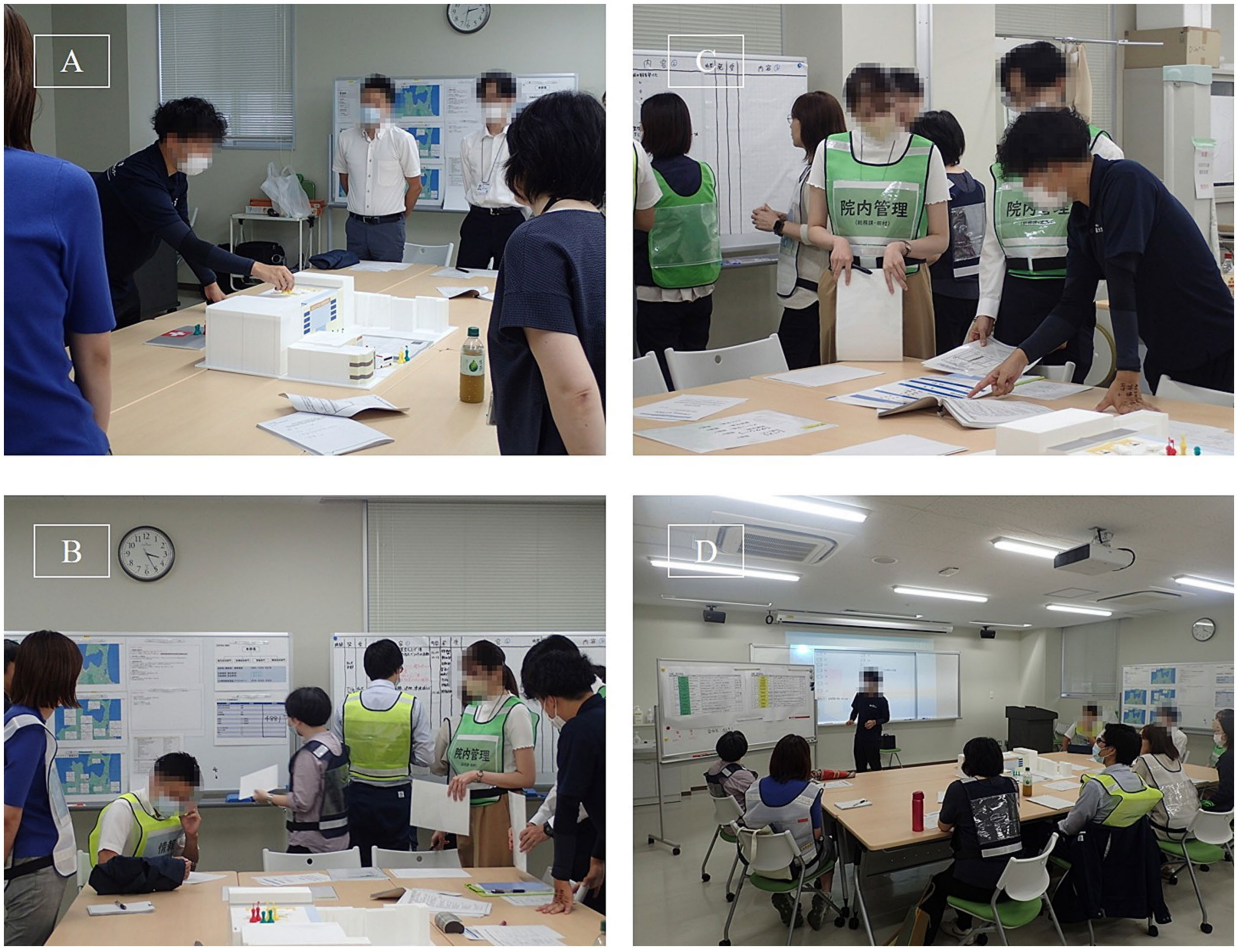
Training session on the BCP assuming a nuclear disaster. **(A)** Lecture on the hospital’s disaster response structure as stipulated in the BCP, using a three-dimensional model of the hospital created with a 3D-printer. **(B)** Participants receiving simulated communications from controllers and compiling information during the disaster response headquarters training. **(C)** Participants checking whether the BCP includes appropriate procedures to respond to the scenarios provided by the controllers. **(D)** Debriefing session following the exercise. On the whiteboard at the front is a list of the scenarios presented by the training controllers, and participants discuss whether each scenario was handled appropriately.

The actual conduct of the disaster response headquarters training is shown in Figure 1. After learning the concepts of establishing a command structure, collecting hazard information, ensuring communication, and conducting regular evaluations as proposed in the Major Incident Medical Management and Support course, a well-known training program for disaster medicine, participants were asked to confirm their roles, particularly in accordance with the hospital’s BCP. In the simulation shown in Figure 1, the controller simulates various roles through telephone communication to impose pressure and simulate the operation of the disaster response headquarters. Subsequently, the participants and instructors shared a one-hour debriefing session, during which they organized their impressions from the simulation and discussed the issues identified in the hospital’s BCP.

From 2023 to 2024, four training sessions with the same content were held with 60 healthcare professionals and administrative staff involved in the operation of the disaster response headquarters. During the debriefing following the simulations, the following issues related to the hospital’s BCP were identified:

- Administrative staff responsible for external phone communication often struggled to understand specialized radiation terminology. It is necessary to include explanations of nuclear disaster-related terms in the BCP and educate on nuclear disasters to disaster medical coordinators other than emergency department doctors and nurses.
- The flow for dispatching radiological technologists to perform contamination screening of evacuated residents has not yet been established and will need to be determined in the future.
- The occurrence of radiation/exposure-related injuries is likely to be of interest to the media. However, media responses are not addressed in the BCP.

## 5 Discussion

These issues appear to have stemmed from several underlying causes. First, the limited familiarity of administrative staff with radiation-related terminology suggests a gap in prior education and training specific to nuclear disasters, which are not commonly addressed in routine hospital disaster preparedness. Second, the absence of a clear operational flow for dispatching radiological technologists likely reflects the fact that contamination screening is not typically included in general hospital emergency response protocols. Finally, the lack of procedures for media response may be due to an underestimation of public and media interest in radiation-related incidents, a topic not widely covered in current BCPs. These insights were discussed and shared during the debriefing sessions and have highlighted the need to revise the BCP to better incorporate scenarios specific to nuclear disasters.

The present study highlights the importance of developing and conducting operational training based on hospital BCP that in corporate nuclear disaster scenarios. The critical role of resilient health systems in disaster preparedness and response has been extensively documented (9), emphasizing the necessity of scenario-based exercises to enhance hospital functionality during complex disasters.

Comprehensive hospital disaster preparedness, which includes detailed BCP and regular training, has been demonstrated to improve readiness and mitigate functional failures during emergencies (10). Communication difficulties and gaps in specialized radiation-related knowledge identified in the current findings align with previously reported challenges related to knowledge deficits in hospital preparedness for infectious and hazardous events (11).

Tailored training according to specific disaster types is essential to equip participants with relevant skills and knowledge (12). The integration of nuclear disaster components into existing BCP and the implementation of operational drills in this study reflect this approach, which is supported by international guidelines and systematic reviews (13).

Clear communication pathways and defined roles are crucial for effective disaster response operations. Hospitals with established role definitions and communication protocols have shown better performance during disaster situations. Furthermore, continuous revision of BCP based on practical training outcomes is essential, as highlighted in the literature on medical and public health preparedness (1). Overall, these findings reinforce that hospital disaster preparedness should be a dynamic, evolving process, incorporating lessons learned from drills and real events to address operational challenges effectively.

Based on the findings of this study, several recommendations can be made to improve future trainings and BCP revisions. First, nuclear disaster terminology should be clearly defined and included in the BCP, and targeted educational sessions should be conducted for administrative staff who may be involved in external communications. Second, a standardized operational protocol for dispatching radiological technologists needs to be developed, ensuring their timely involvement in contamination screening of evacuees. Third, specific guidelines for media response during radiation-related incidents should be incorporated into the BCP. Lastly, continued implementation of simulation-based training followed by structured debriefing will be essential to identify and address emerging issues in a timely manner.

## 6 Conclusion

In Japan, following the Fukushima Daiichi Nuclear Power Plant accident, the nuclear disaster medical system was reviewed and healthcare facilities across the country were required to formulate BCPs for nuclear disaster response. This study outlines the formulation of the BCP at our institution and the associated disaster response headquarters training and reports the challenges identified through training that contributed to the revision of the BCP.

## Data Availability

The raw data supporting the conclusions of this article will be made available by the authors, without undue reservation.
